# Citrus fruits are rich in flavonoids for immunoregulation and potential targeting ACE2

**DOI:** 10.1007/s13659-022-00325-4

**Published:** 2022-02-14

**Authors:** Wenting Liu, Weikang Zheng, Liping Cheng, Ming Li, Jie Huang, Shuzheng Bao, Qiang Xu, Zhaocheng Ma

**Affiliations:** 1 College of Horticulture and Forestry Sciences, Key Laboratory of Horticultural Plant Biology, Ministry of Education, Huazhong Agricultural University, Wuhan, 430070 China; 2 Network of Aquaculture Centres in Asia-Pacific, Bangkok, 10903 Thailand; 3 China Association of Agricultural Science Societies, Beijing, 100125 China

**Keywords:** Citrus flavonoids, Naringin, Immunoregulation, ACE2, SARS-CoV-2

## Abstract

The most recent outbreak of 2019 novel coronavirus, named as COVID-19, caused pneumonia epidemic in Wuhan with 2121 deaths cases as of February 20th 2020. Identification of effective antiviral agents to combat the novel coronavirus is urgently needed. Citrus fruit peel or wild citrus are rich in flavonoids, and clinically documented for roles in relief of cough and promotion of digestive health. Therefore, citrus fruits are assumed to possess antivirus activities or enhance the host immunity. A previous study found that hesperetin could act as a high potent inhibitor of SARS-CoV 3CLpro. We determined six flavonoid compounds’ content in three citrus species by using LC–MS technique. The content of naringin and naringenin was at higher levels in pummelo. Hesperetin and hesperidin were highly accumulated in mandarin and sweet orange. The subsequent in vitro and in vivo experiments indicated that naringin could inhibit the expression of the proinflammatory cytokines (COX-2, iNOS, IL-1β and IL-6) induced by LPS in Raw macrophage cell line, and may restrain cytokine through inhibiting HMGB1 expression in a mouse model. The results revealed that naringin may have a potential application for preventing cytokine storm. We simulated molecular docking to predict the binding affinity of those flavonoids to bind Angiotensin-converting enzyme 2 (ACE 2), which is a receptor of the coronavirus. Consideration of the potential anti-coronavirus and anti-inflammatory activity of flavonoids, the citrus fruit or its derived phytochemicals are promising in the use of prevention and treatment of SARS-CoV-2 infection.

**Graphical Abstract:**

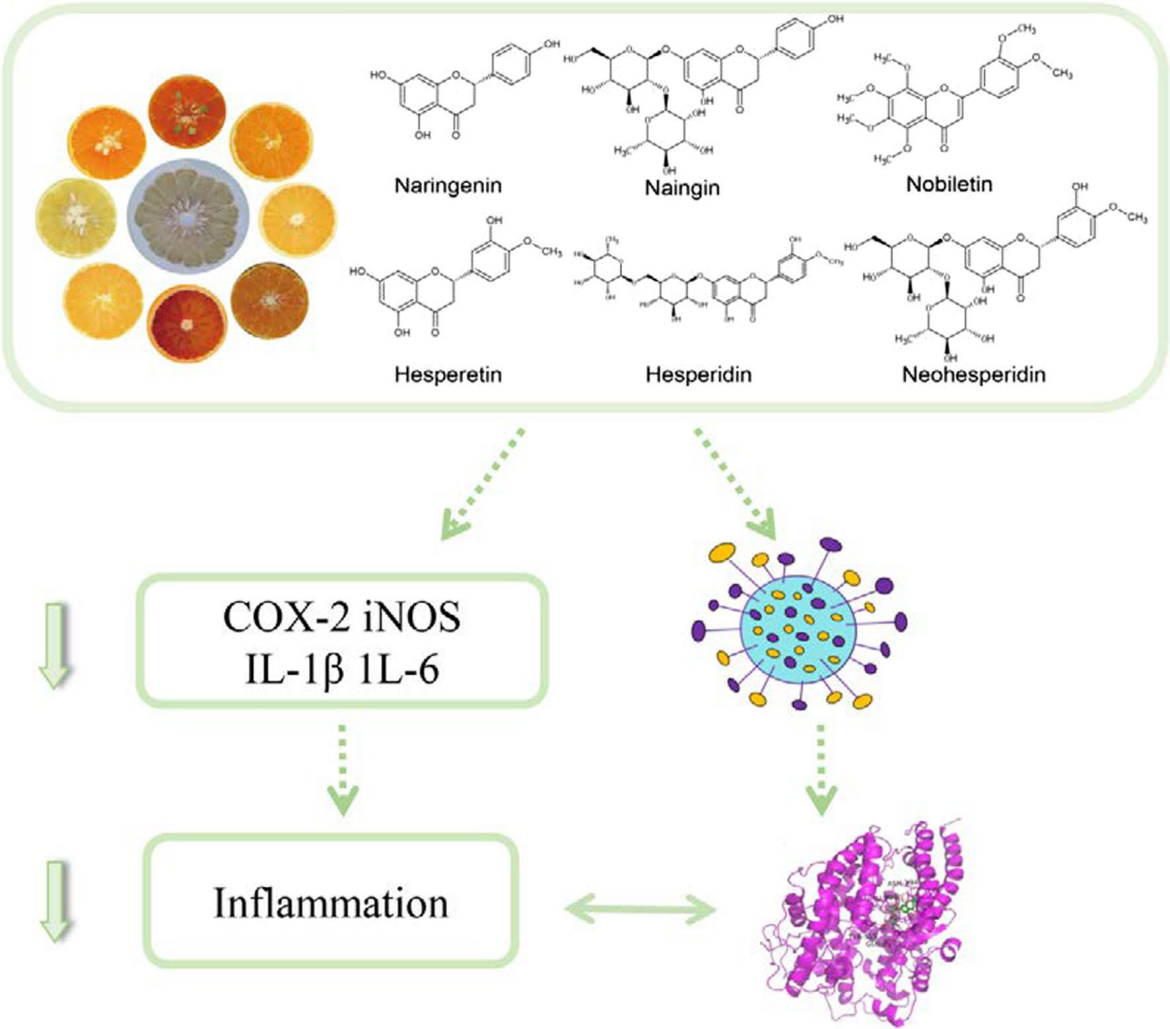

**Supplementary Information:**

The online version contains supplementary material available at 10.1007/s13659-022-00325-4.

## Introduction

The number of pneumonia cases caused by a novel coronavirus (SARS-CoV-2) continues to rise in China. Thus far, there are tens of thousands of confirmed cases by the hospital agency. Common symptoms of patients infected with SARS-CoV-2 are fever, cough and myalgia or fatigue. Structural elucidation of SARS-CoV-2 proteins confirmed that the spikes proteins combined with the cellular receptor ACE2 to achieve various physiological processes. The severe cases have high amounts of cytokines, such as TNF-α, IL-1β, IL-10, IFNγ and MCP-1, suggesting that the cytokine storm is associated with disease severity [1]. Therefore, interrupting the combination of spikes protein to ACE2 is a potential therapeutic target for developing new drugs.

Currently, there is no specific antiviral treatment against the new coronavirus. Identifying effective antiviral agents to combat the disease is urgently needed. Commercial antiviral agents and chemical compounds extracted from traditional Chinese medicinal herbs were screened [2]. Remdesivir and chloroquine were found to be highly effective in the control of SARS-CoV-2 infection in vitro [3]. Some Chinese herbal compounds including baicalin, scutellarin, hesperetin, nicotianamine and glycyrrhizin were predicted to have a capacity for binding ACE2 with potential anti-SARS-CoV-2 effects [4]. The existing safe host-directed therapies were repurposed to treat COVID-19 infection [5]. Traditional Chinese medicine was used to treat the novel coronavirus-infected pneumonia and proved to have a higher effect in the relief of cough and fever-reducing [6]. In addition, corticosteroids were used frequently for severe cases treatment to reduce inflammatory-induced lung injury.

Citrus is rich in bioactive compounds and some varieties are used as Chinese folk medicine, such as Zhiqiao and Zhishi (Sour orange, *Citrus aurantium*) or its hybrids, Huajuhong (*Citrus grandis*), and Chenpi (*Citris reticulata*). They have been clinically documented for roles in the relief of cough and the promotion of digestive health. Flavonoid compounds are expected to be developed as anti-viral drugs. Hesperetin was found the high potent inhibitor of SARS-CoV 3CLpro [7]. The spike proteins (S-protein) of SARS-CoV-2 shared very similar 3-D structures in the receptor-binding domain (RBD) with SARS-CoV. The putative binding activity between molecules could be stimulated by molecular docking software. The ACE2 binding activity of some natural compounds had been estimated by molecular docking [8]. Meanwhile, nutrient supplements could reduce the host immune responses. Therefore, we try to identify effective antiviral and anti-inflammation compounds from citrus flavonoids and give a nutritional recommendation for the prevention and treatment of COVID-19.

## Results and discussion

### Flavonoid profiling in citrus species

**Fig. 1:**
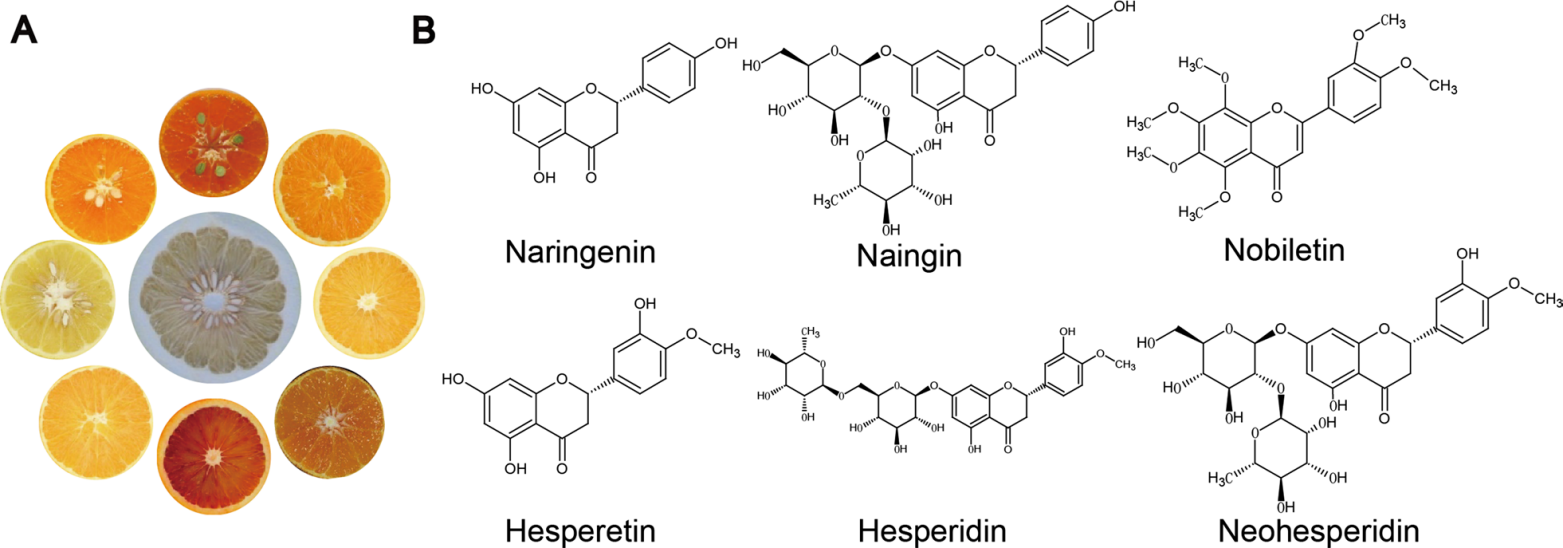
Citrus fruits (**A**) and chemical structure of naringenin, naringin, nobiletin, hesperetin, hesperidin, and neohesperidin (**B**)

**Fig. 2:**
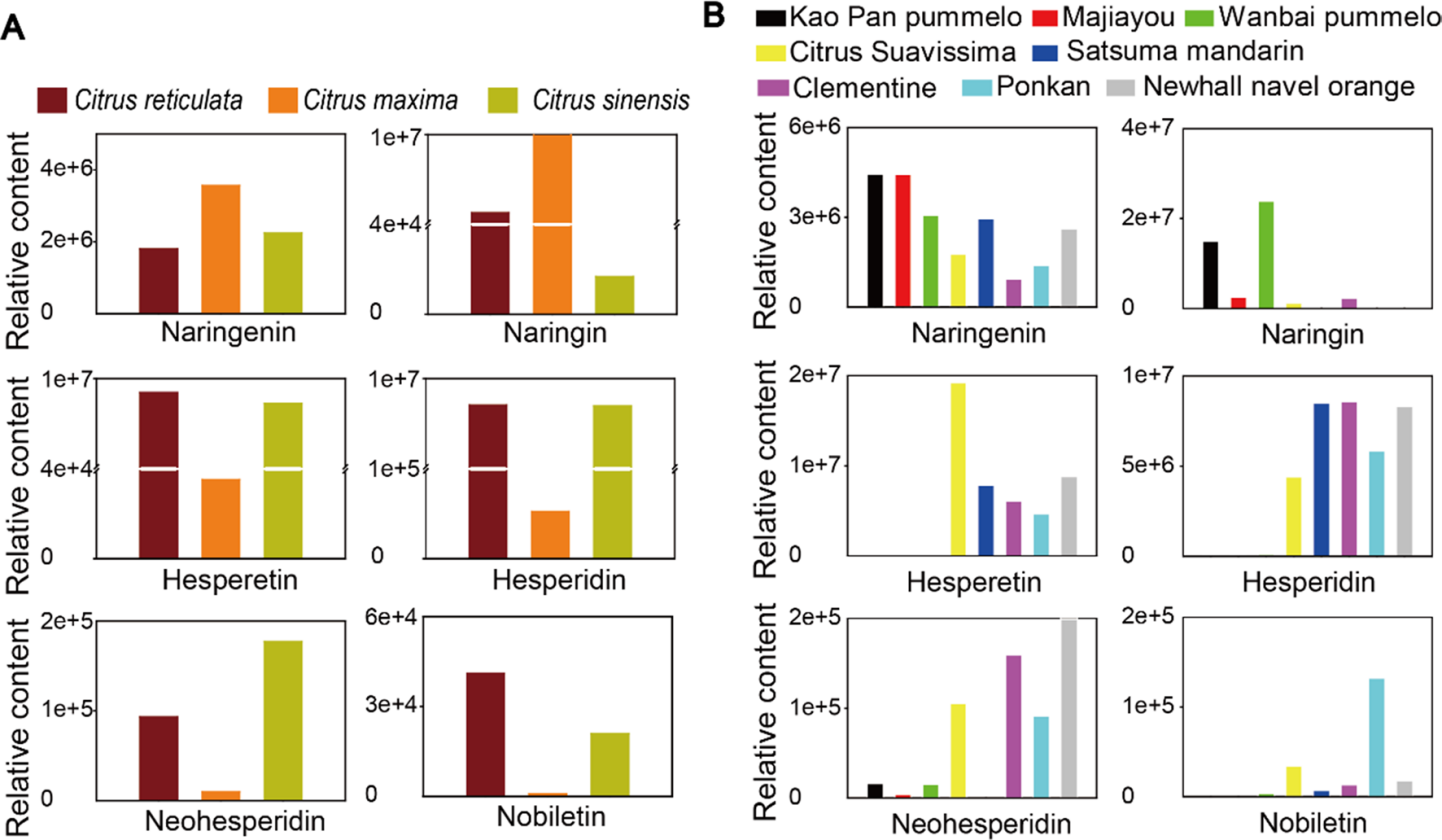
The content of six compounds were analyzed by LC–MS/MS in different citrus species and cultivars. The data were analyzed by software, Lab Solution Insight LCMS. Peak area of ions signal repreasent relative content. **A** Distribution of naringenin, naringin, hesperetin, hesperidin, neohesperidin and nobiletin in different citrus species. **B** The content of six flavonoid compounds in different cultivars

Citrus fruits are a rich source of vitamins, flavonoids and alkaloids, especially the content of flavonoids were richest. These phytochemicals have been reported to benefit human health, used to prevent and treat some diseases [9, 10]. Naringenin, naringin, hesperetin, hesperidin, neohesperidin and nobiletin are the major active constituent of citrus fruits. To assess the potentials of six flavonoid compounds (Fig. 1) variation in citrus, different cultivars were collected from three major species of mandarin (*Citrus reticulata*), pummelo (*Citrus maxima*) and sweet orange (*Citrus sinensis*) for targeted metabolic profiling. We have been detected and quantitated 138 annotated flavonoids in the flesh, including segment membrane and juice sacs of 16 cultivars using LC–MS/MS (Additional file 1: Tables S2 and S3). Among these metabolites, we mainly analyzed the above six flavonoid compounds. The intensity of metabolic profiling signals for total ions reflected substantial qualitative and quantitative differences in different species. The relative content was represented by the average of relative contents of mandarins, pummelos, sweet oranges, respectively. Sixteen cultivars divided into mandarin, pummelo, sweet orange, were compared with each other. As shown in Fig. 2A, the contents of naringin and naringenin were at higher levels in pummelo. On the other hand, mandarin and sweet oranges had higher hesperetin, hesperidin, neohesperidin and nobiletin contents compared to pummelo. Meanwhile, these six flavonoids were detected in selected 8 citrus cultivars, including ‘Kao Pan’ pummelo, ‘Majiayou’ pummelo, ‘Wanbai’ pummelo, ‘Oukan’ mandarin (Citrus Suavissima), Satsuma mandarin, Clementine mandarin, ‘Ponkan’ mandarin, ‘Newhall’ navel orange. As shown in Fig. 2B the content of naringenin and naringin were higher in ‘Kao Pan’ pummelo and ‘Wanbai’ pummelo than other cultivars. Herbal medicine Huajuhong, made from pummelo, also had a high content of naringin. Hesperetin, hesperidin and neohesperidin accumulated higher in Citrus Suavissima, Clementine, Ponkan and ‘Newhall’ navel orange. Therefore, herbal medicine chenpi, made from mandarin, and Zhiqiao, made from a hybrid of mandarin and pummelo (sour orange), had high contents of hesperidin and neohesperidin, respectively. The content of nobiletin accumulated higher in Ponkan. Vitamins, rutin and herbal medicine Chenpi, Zhiqiao and Huajuhong were used in the treatment of SARS-CoV-2 infection. In order to prevent and decrease plasma cytokines levels of TNF-α, IL-1β, IL-10, IFNγ in COVID-19, citrus fruit or its derived phytochemicals should be an option to reduce the host immune responses and prevention of infection.

### Anti-inflammation of citrus naringin in vitro and in vivo

Cytokine storm was observed in most severe COVID-19 patients with increased plasma concentrations of TNF-α, IL-1β, IL-10, and IFNγ. Corticosteroids were used frequently for severe cases of treatments to reduce inflammatory-induced lung injury [11]. Therefore, treatment with anti-inflammatory approach is critical to alleviating clinical symptoms related to COVID-19.

**Fig. 3:**
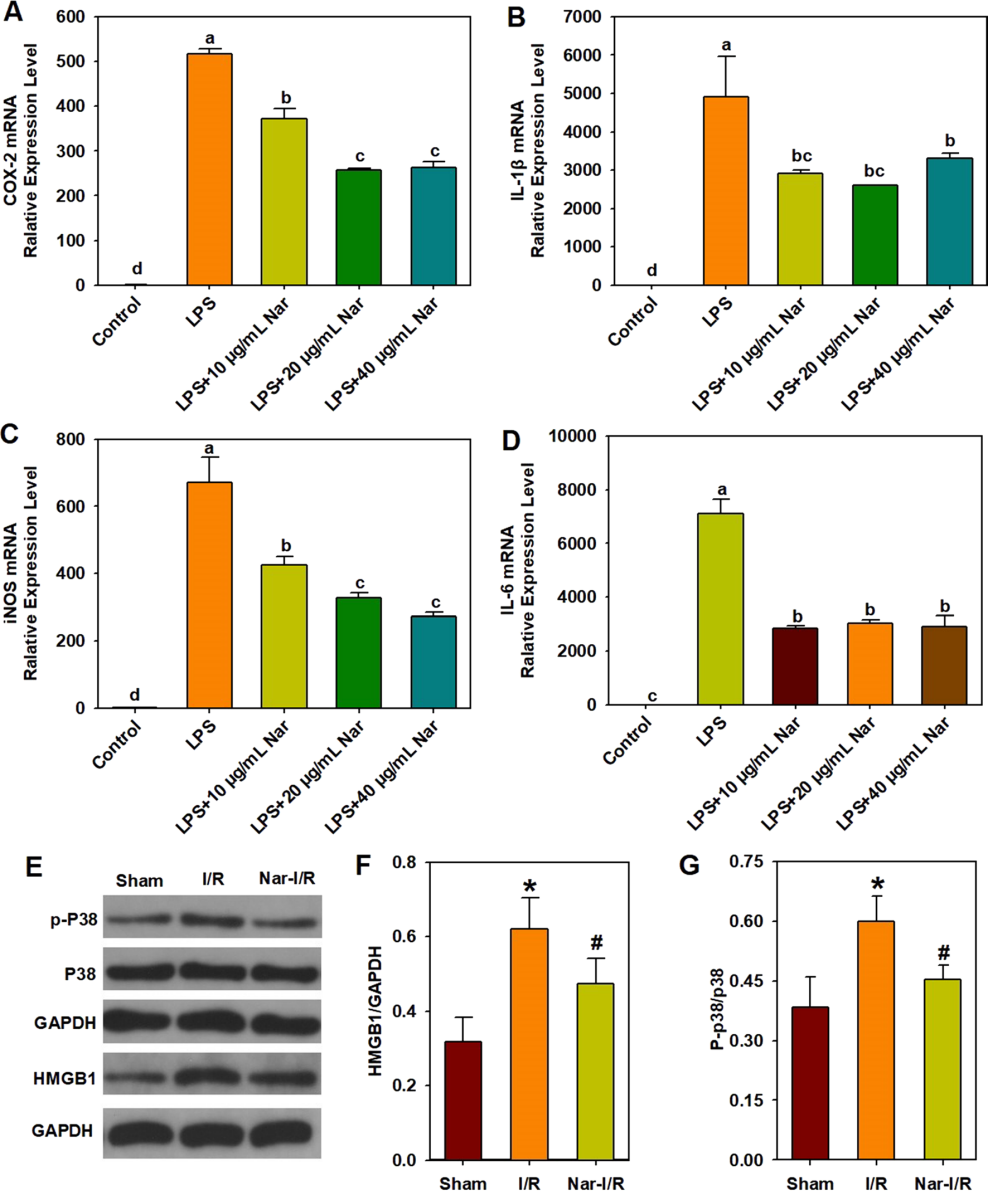
Anti-inflammation of naringin in vitro and in vivo. **A**–**D** Effect of naringin on LPS-induced mRNA expression of COX-2, iNOS, IL-1β, and IL-6 in RAW 264.7 macrophages. The concentration of LPS is 1 μg/mL. **A** COX-2 mRNA relative expression level; **B** IL-1β mRNA relative expression level; **C** iNOS mRNA relative expression level; **D** IL-6 mRNA relative expression level. Different letters show significant differences (*p* < 0.05). **E**–**G** Anti-inflammation effect of naringin on myocardial ischemia/reperfusion injury in rats. **E** Representative images of the Western blot results. **F** HMGB1 protein Expression level; **G** p-P38 protein expression levels. *p < 0.05 vs. the Sham group; ^#^*p* < 0.05 vs. the I/R group. COX-2, cyclooxygenase-2; HMGB1, high mobility group box 1 protein; IL-1β, interleukin-1β; IL-6, interleukin 6; iNOS, inducible nitric oxide synthase; I/R, ischemia/reperfusion; LPS, lipopolysaccharide; Nar, naringin

LPS, a bacterial Gram-negative endotoxin, can induce cytokine storm with the increase of cytokines, such as IL-1β, TNF-α, IFNγ, IL-6 and MCP-1 [12]. In addition, macrophage, one kind of the immune cells, can be caused by LPS to overzealously produce inflammatory cytokines in immune response. The anti-inflammatory effect of citrus naringin on inflammatory cytokines, COX-2, iNOS, IL-1β and IL-6 were determined at mRNA levels in LPS-induced RAW 264.7 macrophages shown in Fig. 3A–D. The results demonstrated that the *COX-2*, *iNOS*, *IL-1β* and *IL-6* mRNA expression levels in LPS-treated macrophage cells were increased compared to the control group. Application of naringin (10, 20 and 40 μg/mL) significantly diminished the effects of LPS induction of *COX-2*, *iNOS, IL-1β* and *IL-6* expression.

Previous studies demonstrated inflammatory response is one of the important symptoms in myocardial ischemia–reperfusion (I/R) injury process, and it was found that myocardial I/R injury could be reduced by inhibiting inflammatory response through regulating the expression of inflammatory cytokines, such as TNF-α, INF-γand IL-6. Thus, myocardial I/R injury in rats was used to further examine the anti-inflammatory activity of naringin in vivo. HMGB1 is a proinflammatory cytokine in the early stages, and plays a critical role in myocardial I/R. The activation of P38 mitogen-activated protein kinase (MAPK) could be enhanced when myocardial I/R injury occurs. In addition, it was suggested that the P38 MAPK signaling pathway is involved in HMGB1 release. Therefore, the expression of HMGB1 level and the phosphorylation P38 MAPK level related to inflammation in the myocardium were measured to further investigate the anti-inflammatory activity of naringin. As shown in Fig. 3E–G, I/R caused a significant increase in expression levels of HMGB1, and p-P38 proteins compared to the Sham group. Compared with the I/R group, the naringin pretreatment significantly reversed the I/R effects on the expression of HMGB1 and p-P38 proteins.

Naringin exhibits a potent anti-inflammatory activity in the present and previous studies [13]. It could inhibit the expression of the proinflammatory cytokines (COX-2, iNOS, IL-1β and IL-6) induced by LPS in vitro in the present study. Moreover, HMGB1 is a ubiquitous DNA-binding nuclear protein and can be released actively by immune cells, such as macrophages and monocytes, following inflammatory stimulation [14, 15]. HMGB1 also acts as a pro-inflammatory cytokine and regulates cytokine storm, up-regulating cytokines such as TNF-α, IL-6, IL-1β, and IL-8 [1]. It is further demonstrated that naringin could restrain cytokine storm to a certain extent through inhibiting HMGB1 expression in this study. Additionally, P38 MAPK has an important role in HMGB1-mediated production of proinflammatory cytokines [16] and previous studies indicated inflammation is related to the phosphorylation level of P38 MAPK [17, 18]. In this study, naringin pretreatment could attenuate p-P38 MAPK level induced by myocardial I/R injury in rats. Thus, our findings further confirmed that naringin could inhibit inflammatory response by repressing inflammatory cytokine expression and downregulating signaling pathway. These results revealed that naringin has potential use for preventing cytokine storm of COVID-19.

### Molecular docking result of citrus flavonoids to ACE2 enzyme, a receptor of the coronavirus

**Fig. 4:**
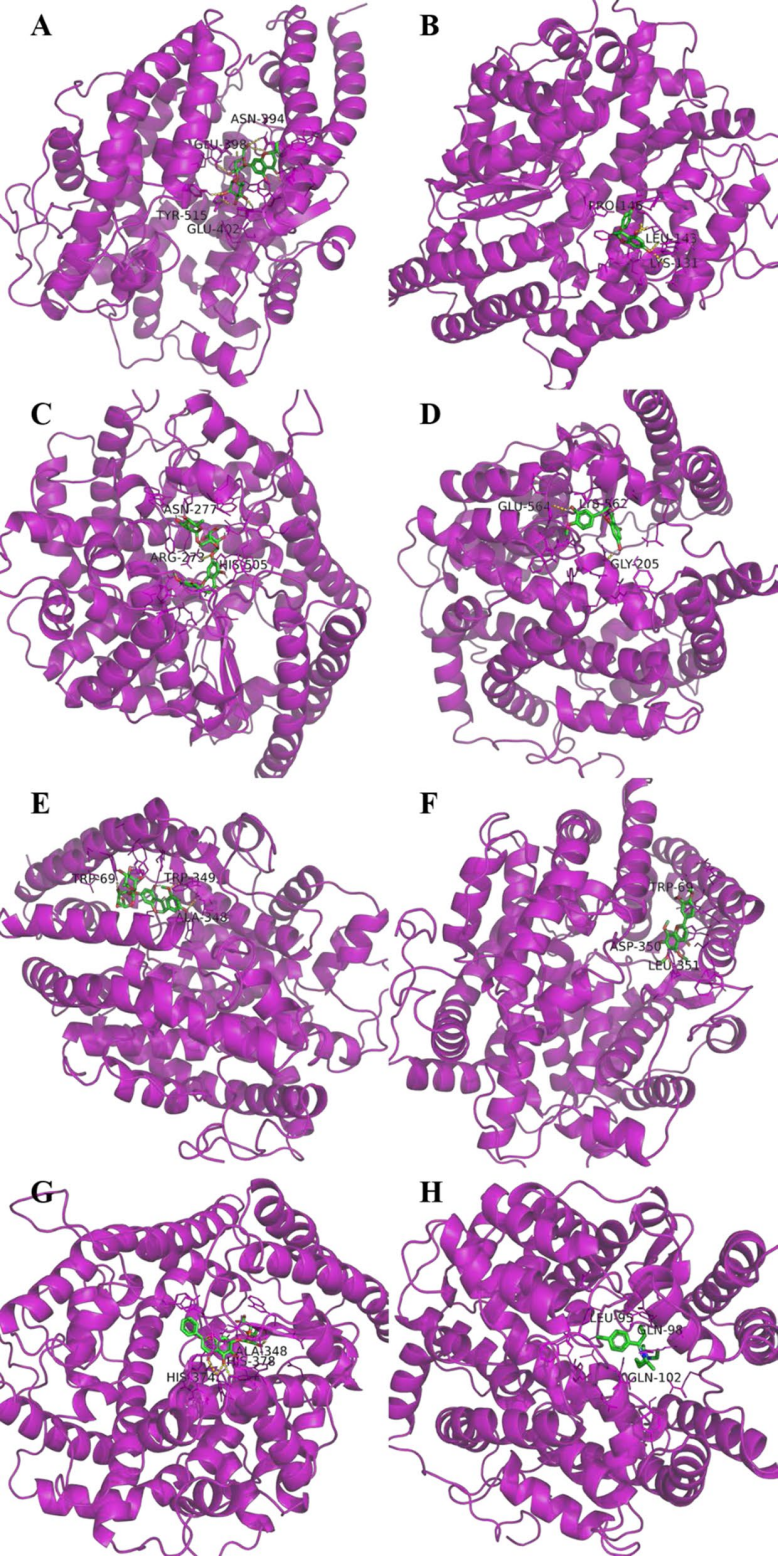
Molecular docking results of naringin (**A**), naringenin (**B**), hesperidin (**C**), hesperetin (**D**), neohesperidin (**E**), nobiletin (**F**), baicalin (**G**), and glycyrrhizin (**H**) to ACE2 enzyme (PDB code: 6ACG). The AutoDock 4.2 software was selected for the docking study using a hybrid Lamarckian Genetic Algorithm (LGA)

In the last few weeks, rapid progress has been made in identification of viral etiology, since one genome sequence (WH-Human_1) of the SARS-CoV-2 was released on Jan 10, 2020. Based on the computer-guided homology modeling method, it is found that SARS-CoV-2 S-protein and SARS-CoV S-protein shared an almost identical 3-D structure in the RBD domain and has a significant binding affinity to human ACE2 [19, 20]. ACE2 is widely expressed in the kidney, lung, brain, digestive tract and is considered to be critical for the coronavirus to enter host cells [21, 22]. Traditional herbal medicine was suggested to be promising for inhibiting coronavirus. Citrus showed broad pharmacological effects, including anti-obesity, anti-oxidant and anti-inflammation [10]. To investigate whether citrus flavonoid compounds have the potential to anti-SARS-CoV-2, we simulated the molecular docking of the six compounds to predict their capacity for binding ACE2 (Fig. 4). The interaction between citrus flavonoids and ACE2 were evaluated by binding energy. The docking result showed that naringin may have the highest binding activity to the ACE2 enzyme with estimated docking energy of − 6.85 kcal/mol, with the potential binding site at TYR-515, GLU-402, GLU-398, and ASN394 (Fig. 4A). Naringenin could bind to ACE2 with estimated docking energy of − 6.05 kcal/mol, with binding site PRO-146, LEU-143, and LYS-131 (Fig. 4B). As shown in Fig. 4C, the stimulated result showed that hesperidin had the potential binding to ACE2 with docking energy of − 4.21 kcal/mol, with binding sites ASN-277, ARG-273, and HIS-505. The molecular docking of hesperetin to the ACE2 enzyme showed that hesperetin had the potential binding to ACE2 with docking energy of − 6.09 kcal/mol, with binding sites LYS-562, GLU-564, GLY-205 (Fig. 4D). Neohesperidin could bind to ACE2, with docking energy of -3.78 kcal/mol, with binding sites at TRP-349, ALA-348, TRP-69 (Fig. 4E). Nobiletin could bind to ACE2 enzyme, with docking energy of − 5.42 kcal/mol, and the potential binding site at TRP-69, LEU-351, ASP-350 (Fig. 4F). These results suggested that among the six citrus flavonoids, the energy required for the binding between naringin and ACE2 was the lowest, followed by hesperetin, narigenin, indicating that they were easier to binding ACE2.

In addition, chloroquine and baicalin had been reported as potential inhibitors of SARS coronavirus infection in vitro test [23, 24]. The cell surface expression of under-glycosylated ACE2 was inhibited by chloroquine, resulting in its poor affinity to SARS-CoV spike protein. We used molecular docking to calculate ACE2 binding energy of chloroquine and baicalin. As shown in Fig. 4H, chloroquine had the potential binding to ACE2 with docking energy of − 5.70 kcal/mol, and binding sites LEU-95, GLN-58, GLN-102. Baicalin could bind to ACE2 enzyme, with docking energy of − 4.70 kcal/mol, with the potential binding site at HIS-374, HIS-378, and ALA-348 (Fig. 4G). The docking energy of naringin, hesperetin and narigenin binding to ACE2 were comparable with chloroquine. It’s worthwhile to conduct further experiments to verify whether these citrus flavonoids could target ACE2 and prevent the infection of SARS-CoV-2 in cell culture models and laboratory animals.

## Materials and methods

### Drugs and reagents

HPLC-grade acetonitrile, acetic acid and methanol were purchased from Merck (Darmstadt, Germany); water was purified with a MilliQ ULTRA purification system (Millipore, Vimodrone, Italy). Authentic standards were provided by Sigma-Aldrich Co. (St. Louis, MO, USA) (https://www.sigmaaldrich.com/china-mainland.html). Standard stock solutions of all metabolites were prepared in methanol. All stock standard solutions were stored at − 20 ℃ in darkness. Naringin (purity > 99%) was isolated from Zhique (*Citrus wilsonii* Tanaka) fruts. Lipopolysaccharide (LPS; from *Escherichia coli* O111:B4) and dimethylsulfoxide (DMSO) were purchased from Sigma (Saint Louis, MO, USA). The Dulbecco’s Modified Eagle’s Medium (DMEM) was obtained from (Hyclone, GE Healthcare, Little Chalfont, UK). Fetal bovine serum (FBS) was purchased from GIBCO-Thermo Fisher Scientific (Waltham, MA, USA). Penicillin and streptomycin were obtained from Hyclone (GE Healthcare, Little Chalfont, UK). Cell culture grade phosphate buffered solution (PBS, pH7.4) were got from Zoman Biotechnology CO., Ltd (Beijing, China). The TRIpure reagent for total RNA extract was bought from Aidlab Biotechnologies Co., Ltd (Beijing, China), the reagents for synthesis of cDNA were purchased Vazyme Biotechnology Co., Ltd (Nanjing, China), and qPCR mix reagents were obtained from Yeasen Biotechnology Co., Ltd (Shanghai, China).

### Sample preparation, extraction, and LC–MS/MS analysis of metabolites of citrus

To study the citrus metabolome, citrus fruits of different cultivars were analyzed. Fruits were collected for analyses at the commercial mature stage and the species were as follows: pummelo (*Citrus maxima*), sweet orange (*Citrus sinensis*), and mandarin (*Citrus reticulata*) (Additional file 1: Table S1). Cultivars were harvested randomly from trees in many positions. The fleshes were separated with sterilized scalpels, then placed in liquid nitrogen and stored at – 80 ℃.

A 100 mg powder was weighed and extracted overnight at 4 ℃ with 1.0 mL 70% aqueous methanol. Following centrifugation at 10,000*g* for 8 min, all of the supernatants were pooled and filtrated (SCAA-104, 0.22 l m pore size; ANPEL, Shanghai, China, http://www.anpel.com.cn/) before LC–MS analysis. The sample extracts were analyzed using an LC–ESI–MS/MS system (HPLC, Shim-pack UFLC SHIMADZU CBM20A system, http://www.shimadzu.com.cn/; MS, Applied Shimadzu LCMS-8060, https://www.shimadzu.com.cn/). The analytical conditions were as follows, solvent system, water (0.04% acetic acid): acetonitrile (0.04% acetic acid); gradient program, 95:5 V/V at 0 min, 5:95 V/V at 12.0 min, 5:95 V/V at 13.2 min, 95:5 V/V at 13.3 min, 95:5 V/V at 15.0 min; flow rate, 0.4 mL/min; temperature, 40 °C; and injection volume: 2 µl. The effluent was alternatively connected to an ESI- triple quadrupole (QQQ)-MS. Quantification of metabolites was carried out in the multiple reaction monitoring (MRM) mode using LC-QQQ- MS/MS.

### Measurement of anti-inflammatory effect of naringin in vitro

The RAW 264.7 macrophage cell line was purchased from Procell Life Science & Technology Co., Ltd (Wuhan, China). The cells were grown in DMEM with high glucose (4.5 g/L) containing 10% fetal bovine serum supplemented with 1% penicillin and streptomycin at 37 °C and 5% CO_2_—95% air in humidified condition. The RAW 264.7 macrophages were treated with LPS (1 μg/mL) or co-treated with LPS (1 μg/mL) and naringin at different concentration (10, 20, 40 μg/mL) for 18 h. To measure the mRNA expression of cyclooxygenase-2 (COX-2), Interleukin-1β (IL-1β), inducible nitric oxide synthase (iNOS) and IL-6, total RNA was isolated from these cell samples according to the manufacturer’s instruction of TRIpure reagent, and then the first-stranded cDNA was synthesized using a reverse transcription kit. Quantitative real time polymerase chain reaction (qPCR) was performed in an ABI 7500 Real-Time System with the SYBR Green PCR Master Mix. Reactions were initiated with an initial incubation at 50 °C for two minutes and 94 °C for ten minutes, followed by 40 cycles of 94 °C for 5 s, 60 °C for 15 s, and 72 °C for 10 s. The relative gene expression levels were calculated using the 2^−ΔΔCt^ method. The gene primers for qPCR are provided in Table 1. The β-actin was used as an internal reference gene between different samples.

**Table 1:** Primer sequences used in the qPCR analysis

Gene name	Forward/reverse primer sequence (5′–3′)
*β-actin*	AGGCTGTGCTGTCCCTGTATGC ACCCAAGAAGGAAGGCTGGAAA
*COX-2*	ATCTGGCTTCGGGAGCACAAC GAGGCAATGCGGTTCTGATACTG
*IL-1β*	GTTGACGGACCCCAAAAGAT CCTCATCCTGGAAGGTCCAC
*iNOS*	GAATCTTGGAGCGAGTTGTGGA GTGAGGGCTTGGCTGAGTGAG-3
*IL-6*	ACAAAGCCAGAGTCCTTCAGA TCCTTAGCCACTCCTTCTGT

### Measurement of anti-inflammatory activity of naringin in vivo

All protocols were approved by the Institutional Animal Care and Use Committee of Wuhan University (Approval Number: 2015-0563) and performed following the Guideline for the Care and Use of Laboratory Animals published by the US National Institutes of Health (NIH Publication, revised 1996). Male Sprague–Dawley rats weighing 200–250 g were purchased from the Animal Experiment Center of Wuhan University (Wuhan, China). The myocardial ischemia/reperfusion injury model was generated and utilized as previously described. Eighteen male Sprague–Dawley rats were randomly assigned to three groups as following: Group 1. Sham-operated control group (Sham) (n = 6): The rats were subjected to surgical manipulation without ligation of the left anterior descending coronary artery (LAD). The rats were treated with solvent (sterile saline with 2% DMSO) for naringin by intravenous (i.v.) injection 10 min before the surgical manipulation; Group 2. Ischemia/reperfusion group (I/R) (n = 6): the rats were subjected to LAD occlusion for 30 min followed by reperfusion for 4 h. After being anesthetized, the rats were treated with solvent for naringin (i.v.) 10 min before LAD occlusion; Group 3. Naringin + I/R group (Nar-I/R) (n = 6): the rats were subjected to LAD occlusion for 30 min followed by reperfusion for 4 h. After being anesthetized, the rats were treated with naringin 4 mg/kg body weight (BW) 10 min before LAD occlusion. At the end of the experiment, animals were euthanized with 22.5 mg/kg BW sodium pentobarbital (i.p.), and the hearts were removed for further assessments. The change of heart protein expression was analyzed by Western blot. The antibody against-high mobility group box 1 (HMGB1) (1:300) and anti-glyceraldehyde-3-phosphate dehydrogenase (GAPDH, 1:1000) were received from the Boster, Wuhan, China. Anti-P38 (1:1000), and anti-p-P38 (1:1000) were obtained from Cell Signaling Technology, Danvers, USA.

### Molecular docking

Molecular docking procedures have been widely used to analyze interactions and binding sites between macromolecules and their ligands. AutoDock 4.2 software (Olson Laboratory, La Jolla, CA) was used for molecular docking of the components into target proteins by Lamarckian genetic algorithm (LGA) in this study. The docking was performed using identified proteins as rigid receptor molecule, whereas naringin, naringenin, hesperidin, hesperetin, neohesperidin, nobiletin were treated as flexible ligands. Finally, according to the AutoDock scoring function, the lowest energy docked conformation was selected as the most probable binding conformation. The PDB structures of all identified proteins were downloaded from the Protein Data Bank (http://www.rcsb.org/pdb).

### Statistical analysis

SPSS 19.0 was used for data analysis. All data are expressed as mean ± SD. Student’s t-test was used for comparison between two groups. Multiple comparisons among groups were evaluated by one-way ANOVA or a Welch test. Student–Newman–Keuls or Dunnett’s T3 test was used for post hoc multiple comparisons. A *P* value < 0.05 was considered statistically significant.

## Conclusion

We determined the contents of six flavonoid compounds in three citrus species by using LC–MS technique. The contents of naringin and naringenin were at higher levels in pummelo. On the other hand, mandarin and sweet oranges had higher hesperetin and hesperidin contents compared to pummelo. The contents of neohesperidin and nobiletin were lower than other four compounds in citrus. Our data also showed that naringin could inhibit the expression of the proinflammatory cytokines (COX-2, iNOS, IL-1β and IL-6) induced by LPS in vitro. It was further demonstrated that naringin could restrain cytokine through inhibiting HMGB1 expression in a myocardial ischemic/reperfusion injury model. The results suggested that naringin could have the potential to prevent cytokine storms of COVID-19. The molecular docking result predicted that naringin and hesperetin had stronger binding affinity the ACE2. We suggested that these two phytochemicals, e.g., naringin and hesperetin are most potential compounds targeting ACE2 receptor, which could prevent coronavirus infection. Chinese traditional medicine is playing an important role in the treatment of COVID-19. We should pay more attention to natural compounds from citrus and other herbal medicine to combat coronavirus in the future.

## Supplementary Information

**Additional file 1: Table S1.** The information of citrus variety used in this study. **Table S2.** The list of metabolites detected in this study. **Table S3.** Accumulation of metabolites in various species.

## Data Availability

All the data supporting the findings of this study are available within the article and/or its supplementary materials.
